# Draft Genome Sequences of Clinical Respiratory Isolates of Mycobacterium goodii Recovered in Ireland

**DOI:** 10.1128/MRA.00531-21

**Published:** 2021-08-05

**Authors:** Simone Mok, Peter R. Flanagan, Emma Roycroft, Thomas R. Rogers, Margaret M. Fitzgibbon

**Affiliations:** a Irish Mycobacteria Reference Laboratory, St. James’s Hospital, Dublin, Ireland; b Department of Clinical Microbiology, Trinity College Dublin, Dublin, Ireland; University of Maryland School of Medicine

## Abstract

Here, we describe the draft genomes of five Mycobacterium goodii isolates that were recovered from respiratory clinical specimens in Ireland. Currently, one complete genome and one draft genome exist publicly for M. goodii.

## ANNOUNCEMENT

Nontuberculous mycobacteria (NTM) are ubiquitous environmental microorganisms. With over 190 species identified, they are commonly found in water systems, vegetation, and soil (1). NTM are an important cause of human infections and may cause pulmonary disease that can resemble tuberculosis (2, 3). Of the many NTM, Mycobacterium goodii is an emerging pathogen. First described in 1999, it is a rapidly growing organism that is associated with nosocomial infections and is related to Mycobacterium smegmatis (4). M. goodii is resistant to many forms of decontamination and sterilization and has been reported in cases of infected pacemaker sites and osteomyelitis associated with prosthetic material (3). Moreover, it is usually resistant to clarithromycin and rifampin due to overexpression of the *wag31* gene, resulting in thickening of the peptidoglycan layer, as well as the presence of the *erm* gene, which confers macrolide resistance (5). Therefore, M. goodii presents a unique challenge in health care settings, and potential outbreaks caused by this rare pathogen are yet to be investigated by whole-genome sequencing (WGS).

Between 2011 and 2020, five M. goodii isolates were recovered from bronchoalveolar lavage or sputum samples from patients throughout Ireland. In all instances, samples were processed in the Irish Mycobacteria Reference Laboratory (IMRL), where culture confirmed the genus as Mycobacterium using the GenoType Mycobacterium CM line-probe assay v2.0 (Bruker-Hain Diagnostics, Germany). Further analysis using the GenoType Mycobacterium AS assay v1.0 confirmed the species as M. goodii.

For WGS, each isolate was cultured in a Bactec MGIT 960 system according to the manufacturer’s instructions (Becton, Dickinson and Company, Franklin Lakes, NJ, USA). Prior to DNA extraction, cultures were heat inactivated for 15 min at 95°C. Following this, genomic DNA extraction was performed using the Fuji QuickGene DNA tissue kit S (Kurabo, Osaka, Japan) according to the manufacturer’s guidelines. Paired-end libraries were prepared using Nextera XT DNA library preparation kits (Illumina, San Diego, CA, USA), and sequencing was performed on an Illumina MiSeq system using a 2 × 300-bp reagent kit v3 as specified by the manufacturer. Unless stated otherwise, all software used default parameters. The sequenced reads were assessed for quality and trimmed using Fastp v0.20.0 (https://github.com/OpenGene/fastp) (6). Following quality control and trimming, *de novo* assembly was performed using the SPAdes genome assembler v3.14.0 (https://github.com/ablab/spades) (7). Each genome was uploaded to the online open-source software and data platform KBase (8). The platform contains a number of analytical tools, and genome annotation was performed using RASTtk v1.073 (9). Whole-genome single-nucleotide polymorphism (SNP) analysis was performed by mapping sequencing reads to the complete genome sequence of M. goodii X7B using Snippy v3.2 (https://github.com/tseemann/snippy), and the average coverage was assessed using Qualimap (10).

Five M. goodii genomes were assembled from 7,868,173 reads, and all genomes contained a number of contigs of >200 bp; all associated metrics for each sample are presented in Table 1. The mean coverage of sequencing reads mapped to the reference genome was 34×. Whole-genome SNP analysis of our clinical M. goodii isolates and M. goodii strain ST0139456 (GenBank accession number PEBB00000000) revealed an average pairwise distance of >20,000 SNPs, indicating different origins (11).

**TABLE 1:** WGS metrics for M. goodii isolates

Isolate	No. of contigs	G+C content (%)	Contig *N*_50_ (bp)	Total no. of reads	Mapping coverage (×)	Genome size (bp)	Total no. of genes	No. of coding genes	No. of noncoding RNAs	No. of noncoding repeats	No. of noncoding prophages	No. of noncoding CRISPR genes^*a*^	BioSample accession no.	GenBank accession no.
11IE50	202	66.82	232,271	863,356	25	7,459,433	7,734	7,476	50	208	NA^*b*^	NA	SAMN19031642	JAHBON000000000
14IE55	181	66.99	219,682	1,530,444	31	6,998,916	7,104	6,940	51	112	1	NA	SAMN19031643	JAHBOM000000000
16IE56	222	66.89	159,038	2,102,252	43	7,406,805	7,764	7,526	74	145	1	18	SAMN19031644	JAHBOL000000000
19IE239	229	66.87	128,208	1,794,620	37	7,210,549	7,516	7,286	51	178	1	NA	SAMN19031645	JAHBOK000000000
20IE209	438	66.80	78,153	1,577,501	34	7,340,765	7,902	7,452	46	403	1	NA	SAMN19031646	JAHBOJ000000000

a Noncoding CRISPR genes consisted of CRISPR_array, CRISPR_repeat, and CRISPR_spacer.

b NA, not applicable.

This report presents the first draft genomes of respiratory M. goodii isolates from Ireland. The data will serve as a useful reference for WGS studies of M. goodii.

### Data availability.

The draft genome of each isolate was deposited in the NCBI database. The BioProject number is PRJNA727929, with BioSample numbers SAMN19031642 (11IE50), SAMN19031643 (14IE55), SAMN19031644 (16IE56), SAMN19031645 (19IE239), and SAMN19031646 (20IE209). The assembled WGS data were deposited in GenBank with accession numbers JAHBON000000000 (11IE50), JAHBOM000000000 (14IE55), JAHBOL000000000 (16IE56), JAHBOK000000000 (19IE239), and JAHBOJ000000000 (20IE209).
